# An exploratory study of relationships among morphological and mechanical properties of the patellar tendon and patient‐reported outcomes after ACLR using BPTB autograft

**DOI:** 10.1002/jeo2.70700

**Published:** 2026-03-24

**Authors:** Claudia Jean Kacmarcik, Naoaki Ito, Karin Grävare Silbernagel

**Affiliations:** ^1^ Department of Physical Therapy University of Delaware Newark Delaware USA; ^2^ Biomechanics and Movement Science Program University of Delaware Newark Delaware USA; ^3^ Department of Orthopedics and Rehabilitation University of Wisconsin‐Madison Madison Wisconsin USA; ^4^ Badger Athletic Performance Program University of Wisconsin‐Madison Madison Wisconsin USA

**Keywords:** functional recovery, knee function, limb symmetry, musculoskeletal imaging, psychological readiness, tendon remodelling, ultrasound elastography

## Abstract

**Purpose:**

Patellar tendon structural and mechanical properties undergo significant remodelling after anterior cruciate ligament (ACL) reconstruction (ACLR) using a bone–patellar tendon–bone (BPTB) autograft. Tendon properties have been described at different time points after surgery, but their relationship to patient‐reported outcome measures (PROMs) is unclear. The purpose of this investigation was to assess how patellar tendon structure relates to PROMs after ACLR using BPTB autograft.

**Methods:**

Thirty‐four individuals with unilateral ACLR using a BPTB autograft participated in this secondary exploratory analysis. At time points 1, 3–4, and 6–9 months after surgery, PROMs were completed and tendon properties (cross‐sectional area [CSA], thickness, shear modulus, and viscosity) were assessed using B‐mode ultrasound imaging and continuous shear wave elastography. Simple linear regressions evaluated the relationship between Limb Symmetry Index of tendon properties (LSI, [involved ÷ uninvolved] × 100) at each time point and PROM scores at the 6–9‐month time point. Multiple regressions assessed associations between involved limb tendon properties at all three time points and PROMs at 6–9 months, adjusting for age, sex, body mass index (BMI), and time from surgery. *R* ^2^ values for simple linear regressions and *R* ^2^ change values for multiple regressions were interpreted with an alpha level of 0.05.

**Results:**

Shear modulus LSI at 1 month was negatively correlated with Anterior Cruciate Ligament–Return to Sport after Injury (ACL‐RSI) scores at 6–9 months (*p* = 0.022, *R* ^2^ = 0.225, β = −0.20). Viscosity LSI at the 3–4 month time point was positively correlated with Tampa Scale of Kinesiophobia (11‐item) (TSK‐11) scores at 6–9 months (*p* = 0.016, *R* ^2^ = 0.197, β = 0.06). Thickness LSI at 6–9 months was positively correlated with Knee Outcome Survey–Activities of Daily Living Scale (KOS‐ADLS) scores at 6–9 months (*p* = 0.031, *R* ^2^ = 0.156, β = 0.03). When involved limb patellar tendon properties and covariates were considered, no significant relationships with PROM scores were observed (*p* = 0.120–0.960).

**Conclusion:**

Limb symmetry in tendon properties, but not involved limb properties alone, was associated with PROMs 6–9 months after ACLR using BPTB autograft. This suggests that limb symmetry of tendon properties may relate to self‐reported recovery.

**Level of Evidence:**

Level II.

**Abbreviations**
ACL: anterior cruciate ligament
ACLR: anterior cruciate ligament reconstruction
ACL‐RSI: Anterior Cruciate Ligament–Return to Sport after Injury
BPTB: bone–patellar tendon–bone
CSA: cross‐sectional area
cSWE: continuous shear wave elastography
IKDC: International Knee Documentation Committee‐Subjective Knee Evaluation Form
KOS‐ADLS: Knee Outcome Survey–Activities of Daily Living Scale
LSI: Limb Symmetry Index
PROMs: patient‐reported outcome measures
TSK‐11: Tampa Scale of Kinesiophobia (11‐item)

## INTRODUCTION

Approximately 200,000 anterior cruciate ligament (ACL) injuries occur annually in the United States, and most are treated with reconstruction surgery [26]. One of the most common reconstruction techniques uses the bone–patellar tendon–bone (BPTB) autograft, where the central third of the patellar tendon is harvested, inducing a defect through the long axis of the tendon and leaving medial and lateral portions of patellar tendon with a central gap that fills over time [23, 28]. Early in recovery and up to six years after surgery, the graft‐site patellar tendon exhibits greater thickness and cross‐sectional area (CSA) compared to the contralateral limb [4, 19, 33, 35]. One month after surgery, the surgical limb demonstrates a strong relationship between larger patellar tendon CSA and greater quadriceps strength [21]; however, it is unclear if CSA or other patellar tendon metrics relate to additional functional measures used in clinical practice [20].

Patient‐reported outcome measures (PROMs) are used in research and clinical practice to evaluate outcomes and assist with treatment decisions [14, 15, 34]. Function, fear of movement, and readiness to return to sport are a few of the patient characteristics captured by PROMs, assisting clinicians in decision‐making and treatment individualization. In ACL rehabilitation, PROMs are used to quantify patients' subjective functional and psychological readiness to return to sport [5]. For example, scores on PROMs like the ACL‐Return to Sport Index (ACL‐RSI) and International Knee Documentation Committee‐Subjective Knee Evaluation Form (IKDC) differentiated between those who did and did not return to sport 6 months after surgery [25]. Similarly, readiness for advanced ACL rehabilitation at 12 weeks was related to scores on the Tampa Scale of Kinesiophobia (11‐item) (TSK‐11) and IKDC scores 4 weeks after reconstruction [6]. These studies and many others have demonstrated the value of PROMs throughout rehabilitation; however, the relationship between measures of self‐reported recovery and tendon properties has not been studied up to this point.

This exploratory secondary analysis leveraged data from an ongoing, prospective cohort study assessing individuals after ACLR using BPTB autograft and aimed to examine the potential relationship of patellar tendon structure and PROMs. We hypothesize that for each time point, better patellar tendon healing (i.e., more thickness, large CSA) will relate to better 6–9‐month PROM scores.

## METHODS

### Participants

This investigation is an exploratory secondary analysis of a prospective cohort study aimed at assessing recovery after anterior cruciate ligament reconstruction (ACLR) using a BPTB autograft; therefore, a study‐specific power analysis was not performed. Participants were enroled between January of 2021 and December of 2022. Data were collected at time points 1 month, 3–4 months, and 6–9 months after primary ACLR with BPTB autograft. A history of unilateral ACLR using BPTB autograft and age between 13 and 45 years old were the inclusion criteria. Exclusion criteria included a history of ipsilateral or contralateral ACLR, resting heart rate less than 40 or greater than 100 beats per minute, resting blood pressure outside 90/60–180/90 mmHg, or any other symptoms that the physical therapist in charge deemed to be unsafe for participation. Individuals who qualified for study inclusion but were screened more than 1 month after surgery were enroled at the 3–4‐month time point. At each time point, participants completed PROMs assessing past medical history, knee function, and kinesiophobia. A single physical therapist with >2 years of experience performed all assessments of tendon structure and was blinded to PROM scores during acquisition and analysis of structural measures. The University of Delaware Institutional Review Board approved this study (number: 1554102). All adult participants gave informed consent, and individuals under 18 provided assent in addition to the informed consent of their parent or guardian prior to participation.

### PROMs

Four PROMs were collected at the 6–9‐month time point: The IKDC [15], Knee Outcome Survey–Activities of Daily Living Scale (KOS‐ADLS) [16], TSK‐11 [7], and ACL‐RSI [34] (Table 1).

**Table 1:** Descriptions of patient‐reported outcome measures.

Outcome measure	Factors assessed	Score range	Low scores	High scores
International Knee Documentation Committee Subjective Knee Evaluation Form (IKDC) [15]	Symptoms, function, and sports activity	0–100	Low function, high symptoms	High function, low symptoms
Knee Outcome Survey–Activities of Daily Living Scale (KOS‐ADLS) [16]	Knee‐related functional limitations	0–100	Low function	High function
Tampa Scale of Kinesiophobia (11‐item) (TSK‐11) [7]	Fear of movement	11–44	Low fear of movement	High fear of movement
Anterior Cruciate Ligament–Return to Sport after Injury (ACL‐RSI) [34]	Emotions, confidence, and risk appraisal with respect to returning to sport	0–100	Negative psychological response	No negative psychological response

### Tendon structural measures

#### Ultrasound imaging of the patellar tendon

Patellar tendons of both the involved and uninvolved limbs were imaged using B‐mode ultrasound (GE Healthcare) with a 5 cm linear array transducer with gain optimized for tendon identification at a depth of 2.5−3 cm and a frequency of 10 Hz. Participants were positioned in supine with knees in 30° of flexion supported by a foam roller. Images were taken in two planes: along the short axis of the tendon at 50% patellar tendon length using a standoff pad to optimize visualization and along the long axis at the central third of the tendon [18]. Patellar tendon CSA (cm^2^) was measured in the short‐axis images, and in the long axis images, thickness (cm) was measured 1 cm distal to the inferior patellar pole. Three images were taken at each location, and the average measurement from the three images was used for analysis [19]. Excellent intra‐rater reliability has been reported for tendon morphology measurements (intraclass correlation [ICC] = 0.928–0.921) [18].

#### Continuous shear wave elastography (cSWE)

cSWE (Ultrasonix) was used to assess tendon mechanical properties on each limb [10, 11, 32]. Participants were seated with both hips and knees in 90 degrees of flexion with their feet secured in custom boots [18]. Images were taken just distal to the patellar apex in the central region of the patellar tendon while an external actuator delivered shear waves via the quadriceps tendon [10, 11, 20]. Shear modulus (kPa), a tissue's capacity for resisting shear stress and viscosity (Pa‐s), a tissue's ability to resist deformation over time, were calculated using Voight's model for viscoelasticity [9, 10, 11, 20, 31, 32]. For analysis, the average of three trials was used. Good intra‐rater reliability has been reported for both shear modulus and viscosity (ICC = 0.813 and 0.880) [20].

### Statistical analysis

Patient demographics were reported using standard descriptive statistics. Simple linear regressions were used to assess the relationship between limb symmetry in patellar tendon CSA, thickness, shear modulus and viscosity at 1, 3–4, and 6–9 months with 6–9‐month scores on the IKDC, KOS‐ADLS, TSK‐11 and ACL‐RSI. Multiple linear regressions were used to assess the ability of these patellar tendon properties in the involved limb at 1, 3–4, and 6–9 months to predict scores on the same PROMs at 6–9 months.

#### Simple linear regressions using limb symmetry of tendon measures

Limb symmetry indices (LSIs, %, [involved limb ÷ uninvolved limb] × 100) were calculated for tendon structural measures to assess the relationship of tendon structure symmetry at 1, 3–4, and 6–9 months with PROMs at 6–9 months. *R* ^2^ values were calculated and interpreted with an alpha level of 0.05. Previous analyses assessing individuals after ACL injury have demonstrated stability of the uninvolved limb's functional performance and tendon properties throughout recovery, indicating its usefulness as a comparator [2, 19]. Utilizing LSIs minimizes the impact of personal factors such as anthropometrics and individual variability across a sample. Limb symmetry calculations are clinically accessible and interpretable; therefore, tendon LSIs were included in this exploratory analysis.

#### Multiple linear regressions using involved limb tendon measures

In a separate analysis, the relationships between tendon structure in the involved limb and PROMs were evaluated using multiple linear regressions. The base model adjusted for age in years, sex, body mass index (BMI), and the time between surgery and testing (as a continuous variable), due to their influence on tendon morphology, tendon mechanical properties [18, 24, 29], and outcomes after ACLR [12, 17, 22, 36]. Four base models evaluated the ability of age, sex, BMI, and time from surgery to predict PROM scores. For each of these base models, four additional models were evaluated, each including one tendon structure metric (CSA, thickness, shear modulus, or viscosity) as a predictor. Significance (*α* = 0.05) of the *R* ^2^ change was interpreted for each model with tendon structure. Adjustments for multiple comparisons were performed in the case of significant findings.

## RESULTS

### Participants

**Figure 1:**
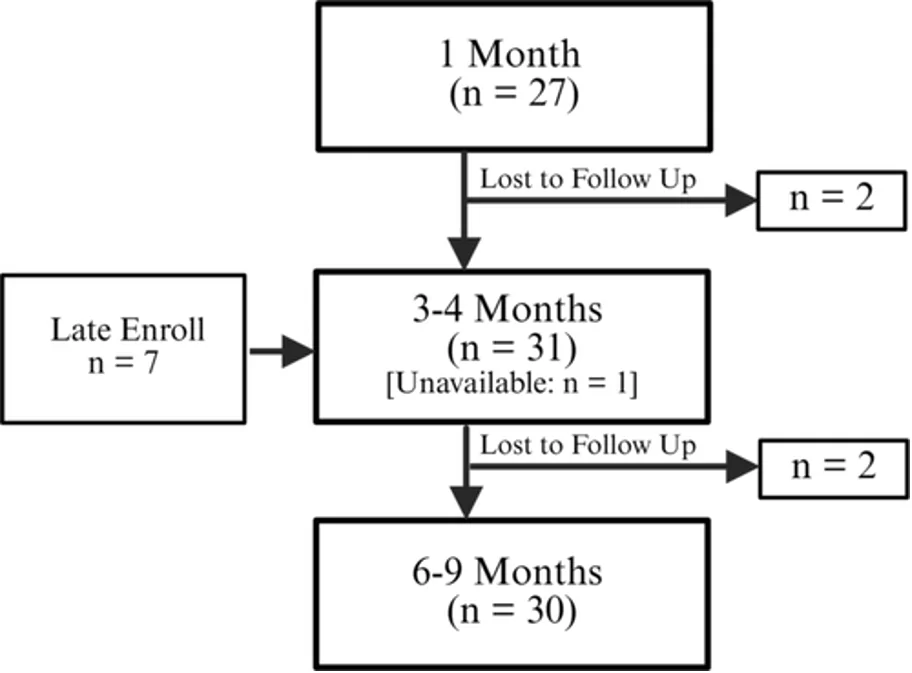
Flowchart of participant enrolment and attrition throughout the study.

Thirty‐four participants (15 female and 19 male) were included in this analysis (Figure 1). Of these, 14 females and 13 males (22.6 ± 7.2 years, BMI: 24.9 ± 3.5 kg/m^2^) were enroled for the 1‐month time point, and 1 additional female and 6 additional males (22.0 ± 4.0 years, BMI: 24.0 ± 2.2 kg/m^2^) were enroled at the 3–4‐month time point. Additional demographic characteristics are outlined in Table 2, and PROM scores and structural measures at each time point are outlined in Table 3.

**Table 2:** Participant demographics by time point.

Measure	1 Month	3–4 Months	6–9 Months
Sex (F:M)	14:13	14:17	13:17
Months since surgery (mean [SD])	1.33 [0.29]	3.42 [0.26]	6.76 [0.67]
Pre‐injury IKDC activity level (I:II:III)^a^	24:2:1	28:2:1	27:2:1

Abbreviations: F, female; IKDC, International Knee Documentation Committee‐Subjective Knee Evaluation Form; M, male; SD, standard deviation.

^a^ Level I: cutting, jumping, pivoting; Level II: lateral movements, less pivoting; Level III: light activities, running.

**Table 3:** Tendon structure, limb symmetry, and PROM scores by time point.

Variable	1 Month	3–4 Months	6–9 Months
	(Mean [SD])	(Mean [SD])	(Mean [SD])
CSA LSI (%)	204.9 [47.2]	190.4 [40.9]	180.0 [41.2]
Thickness LSI (%)	219.7 [54.4]	206.4 [53.0]	189.9 [48.1]
Shear modulus LSI (%)	128.3 [48.7]	107.4 [50.9]	105.7 [51.6]
Viscosity LSI (%)	119.9 [55.0]	107.0 [31.5]	92.1 [43.6]
KOS‐ADLS	65.0 [14.7]	87.7 [7.3]	92.4 [4.1]
IKDC	45.1 [11.8]	65.4 [9.2]	79.9 [8.2]
TSK‐11	22.7 [4.5]	20.6 [3.7]	17.8 [4.4]
ACL‐RSI	59.2 [21.7]	60.2 [20.7]	68.5 [19.8]

Abbreviations: ACL‐RSI, Anterior Cruciate Ligament–Return to Sport Index; CSA, cross‐sectional area; IKDC, International Knee Documentation Committee Subjective Knee Evaluation Form; KOS‐ADLS, Knee Outcome Survey–Activities of Daily Living Scale; LSI, Limb Symmetry Index; PROM, patient‐reported outcome measure; SD, standard deviation; TSK‐11, Tampa Scale of Kinesiophobia (11‐item).

### Relationship between limb symmetry in patellar tendon structure at 1, 3–4, and 6–9 months and PROMs at 6–9 months

**Figure 2:**
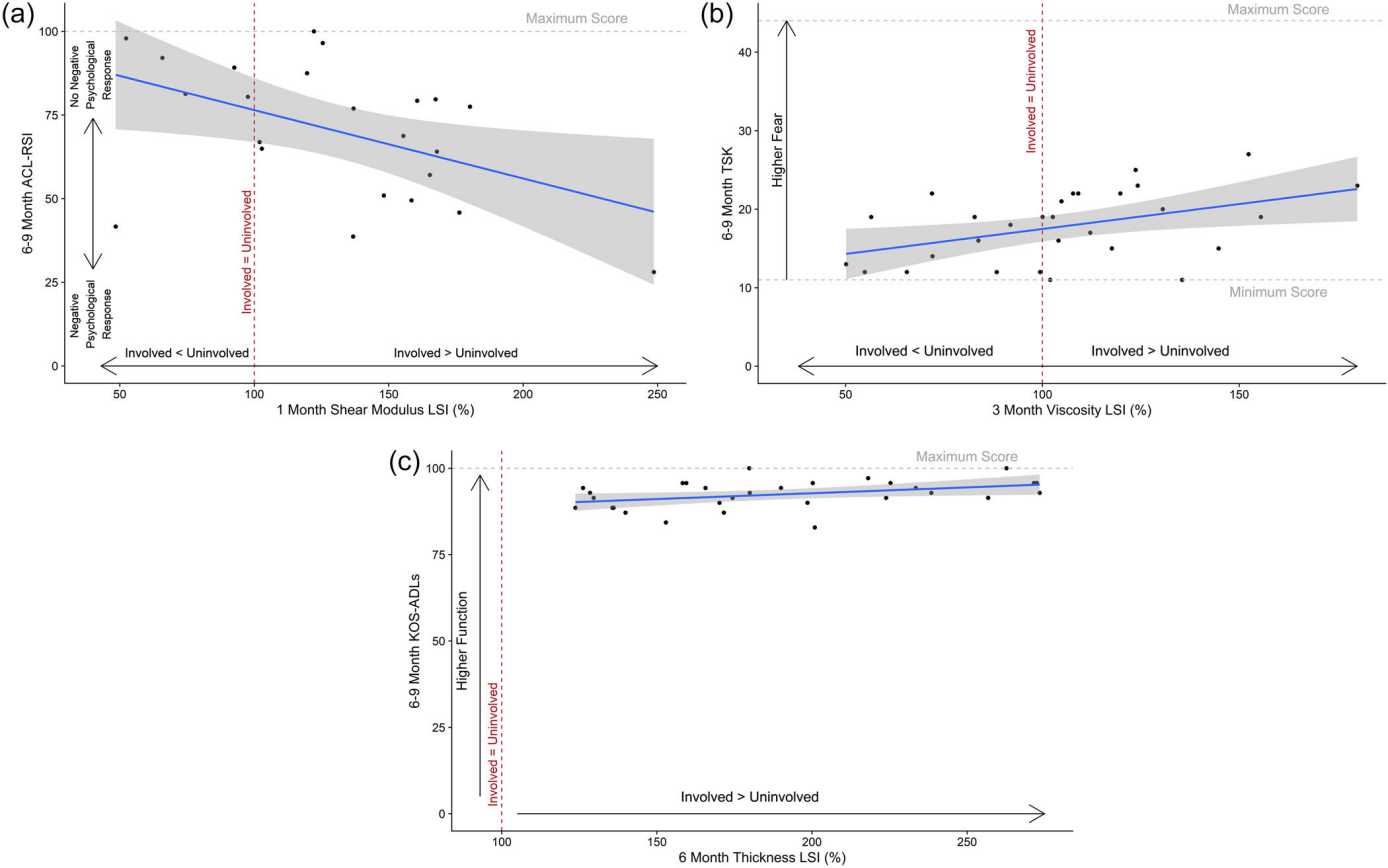
(a–c) Correlations demonstrating significant relationships between PROMs and tendon structural limb symmetry. The blue line indicates the trend of the scatter plot, with the shaded region indicating the 95% confidence interval. Vertical dashed red lines denote 100% limb symmetry. Points to the right of the line indicate the involved limb structure had a greater value than the uninvolved limb. PROMs, patient‐reported outcome measures.

At the 1‐month time point, shear modulus LSI ([involved limb ÷ uninvolved limb] × 100) was negatively correlated with 6–9‐month ACL‐RSI scores (*p* = 0.022, *R* ^2^ = 0.225, β = −0.20), meaning that low shear modulus LSI scores related to better ACL RSI scores (Figure 2a). At the 3–4‐month time point, viscosity LSI was positively correlated with 6–9‐month TSK scores (*p* = 0.016, *R* ^2^ = 0.197, β = 0.06). Large viscosity LSI scores related to higher levels of movement‐related fear (Figure 2b). At the 6–9‐month time point, patellar tendon thickness LSI was positively correlated with 6–9‐month KOS‐ADLS scores (*p* = 0.031, *R* ^2^ = 0.156, β = 0.03), meaning that greater thickness in the involved limb compared to the uninvolved limb (LSI > 100%) related to better self‐reported daily function (Figure 2c). It is important to note that low shear modulus and viscosity in the involved relative to the uninvolved limb (not complete limb symmetry, i.e., 100% LSI), related to better outcomes (Figure 2a,b).

### Involved limb patellar tendon structure at 1, 3–4, and 6–9 months predicting PROMs at 6–9 months

No PROMs at the 6–9‐month time point were significantly related to involved limb patellar tendon thickness, CSA, shear modulus, or viscosity at any time point (1 month, 3–4 months, or 6–9 months, Table 4) after ACLR (overall model *p* = 0.120–0.960) accounting for age, sex, BMI, and time since surgery.

**Table 4:** Multiple regression between involved limb tendon structure at 1, 3–4, and 6–9 months and PROMs at 6–9 months.

Outcome variable (6–9 months)	Predictor variable	1 Month					3–4 Months					6–9 Months				
		Base model *R* ^2^	*R* ^2^	*p* value of model	*R* ^2^ change	*p* value of *R* ^2^ change	Base model *R* ^2^	*R* ^2^	*p* value of model	*R* ^2^ change	*p* value of *R* ^2^ change	Base model *R* ^2^	*R* ^2^	*p* value of model	*R* ^2^ change	*p* value of *R* ^2^ change
KOS‐ADLS	Cross‐sectional area	0.156	0.161	0.67	0.005	0.76	0.064	0.084	0.81	0.021	0.47	0.063	0.179	0.41	0.116	0.08
	Thickness		0.193	0.56	0.037	0.39		0.100	0.75	0.036	0.34		0.153	0.52	0.090	0.12
	Viscosity		0.359	0.15	0.204	0.03		0.064	0.89	0.000	0.95		0.152	0.52	0.089	0.13
	Shear modulus		0.166	0.65	0.010	0.65		0.238	0.23	0.174	0.03*		0.093	0.78	0.030	0.38
IKDC	Cross‐sectional area	0.031	0.031	0.99	0.000	0.93	0.094	0.142	0.56	0.048	0.26	0.106	0.221	0.27	0.115	0.07
	Thickness		0.063	0.95	0.032	0.46		0.112	0.70	0.018	0.49		0.106	0.72	0.000	0.99
	Viscosity		0.291	0.27	0.261	0.02*		0.095	0.77	0.000	0.92		0.111	0.70	0.005	0.72
	Shear modulus		0.378	0.12	0.348	0.01*		0.178	0.42	0.084	0.13		0.106	0.72	0.000	0.95
TSK	Cross‐sectional area	0.108	0.143	0.72	0.036	0.41	0.108	0.112	0.70	0.004	0.75	0.123	0.177	0.42	0.054	0.22
	Thickness		0.108	0.83	0.000	0.94		0.147	0.54	0.038	0.31		0.124	0.64	0.001	0.89
	Viscosity		0.180	0.60	0.073	0.24		0.238	0.23	0.129	0.06		0.123	0.65	0.000	0.98
	Shear modulus		0.178	0.61	0.071	0.24		0.175	0.43	0.067	0.18		0.164	0.47	0.040	0.29
ACL‐RSI	Cross‐sectional area	0.111	0.279	0.30	0.169	0.06	0.013	0.040	0.96	0.027	0.42	0.040	0.040	0.96	0.000	0.97
	Thickness		0.183	0.59	0.072	0.24		0.105	0.73	0.091	0.13		0.138	0.58	0.098	0.11
	Viscosity		0.177	0.61	0.067	0.26		0.013	1.00	0.000	0.99		0.057	0.91	0.017	0.52
	Shear modulus		0.119	0.80	0.009	0.69		0.064	0.89	0.051	0.27		0.102	0.74	0.063	0.21

Abbreviations: ACL‐RSI, Anterior Cruciate Ligament–Return to Sport Index; IKDC, International Knee Documentation Committee Subjective Knee Evaluation Form; KOS‐ADLS, Knee Outcome Survey–Activities of Daily Living Scale; LSI, Limb Symmetry Index; PROM, patient‐reported outcome measure; TSK, Tampa Scale of Kinesiophobia.

* *p* < 0.05.

## DISCUSSION

This exploratory investigation is the first to assess the relationship between patellar tendon structural and mechanical properties and PROMs after ACLR using BPTB autograft. The results demonstrated that limb symmetry in shear modulus at 1 month, viscosity at 3–4 months, and thickness at 6–9 months related to 6–9‐month scores on the ACL‐RSI, TSK‐11, and KOS‐ADLs, respectively. However, when considering only the involved limb and demographic characteristics, tendon properties were not related to PROM scores at the 6–9‐month time point.

A comprehensive clinical picture of tendon health includes the assessment of five domains: symptoms, structure, function, patient‐related factors, and psychological factors [13, 21]. Each domain follows a timeline of healing and recovery that is independent of the other domains. When assessed independently, tendon structure and PROM scores are considered important in clinical practice; however, the results did not demonstrate a direct relationship between the two. Recovery trajectories could help explain these results, as tendon structure, symptoms, and function recover on different timelines [1, 30].

### Limb symmetry in tendon mechanical properties

Weak, positive associations were found between LSI of tendon mechanical properties early after surgery and psychological factors later in recovery. In this study, low stiffness and viscosity LSI 1 month after surgery, not complete limb symmetry (i.e., 100% LSI), was associated with better psychological outcomes at 6–9 months. While the TSK and ACL‐RSI are multifactorial, this analysis did not have sufficient power to control for additional factors, such as physical function, which can impact both psychological and mechanical properties [9, 30, 37]. Since tendon mechanical properties fluctuate throughout recovery [27, 37, 38, 39], and demonstrated relationships were modest (*R* ^2^ = 0.20–0.23), mechanical properties, in addition to other factors like functional and symptomatic outcomes, may be linked to patients' psychological status after ACLR [3, 6, 8].

### Limb symmetry in tendon structural properties

Six to nine months after ACLR, higher function was reported by individuals who, at 1 month, demonstrated a greater degree of tendon thickness compared to the uninvolved limb. Increases in tendon size are a positive response, characteristic of tendon healing [27]. Larger differences in thickness between limbs may be reflective of ongoing tendon remodelling, potentially influenced by personal factors or the frequency and duration of loading throughout key points of recovery. Given the moderate relationship (*R* ^2 ^= 0.16) demonstrated between tendon thickness and functional outcomes, future investigations should seek to determine factors that contribute to optimal tendon remodelling following injury or surgery. While promising, these results should be interpreted with caution, as the lowest observed KOS‐ADLS score at 6–9 months was 83, and two individuals scored the maximum of 100. These high scores represent a ceiling effect, indicating an inability to discriminate among high‐functioning individuals (Figure 2c).

### Involved limb structural and mechanical properties

Tendon properties vary as remodelling occurs throughout recovery. Despite adjusting for age, sex, BMI, and time from surgery, without the uninvolved limb as a reference, the tendon healing that occurred in the first 6 months after BPTB autograft harvest was not predictive of patients' self‐reported function, kinesiophobia, or readiness to return to sport 6 months after surgery [12, 20]. These results align with previous literature demonstrating that recovery in one domain of tendon health may not influence the status of another [1, 8].

## LIMITATIONS

This investigation was an exploratory analysis using data from a parent study, which was powered to detect cross‐sectional differences. Given the exploratory nature of this investigation, an a priori power analysis was not performed. The small sample size available for this secondary prospective analysis limited the statistical power to detect small to moderate effects and increased the risk of Type II error. Future studies with larger sample sizes and statistical power to predict prospective outcomes are required to fully understand these relationships. Participant enrolment and attrition are another limitation of this study, as only 23 of the 34 individuals had complete data at all three time points, preventing a longitudinal analysis. This analysis would also benefit from other assessments of function, given the ceiling effect present in KOS scores. While the observational nature of this study precluded control of other potential confounding variables, such as the rehabilitation protocol and frequency and duration of physical therapy treatment, it enhances the generalizability of findings.

## CONCLUSION

A direct relationship between involved limb tendon structure and PROMs was not observed; however, limb symmetry in tendon properties appears to be part of the clinical picture. Future analyses will inform patient care during recovery after ACLR using BPTB autograft.

## AUTHOR CONTRIBUTIONS

Naoaki Ito and Karin Grävare Silbernagel contributed to the study conception and design. Material preparation, data collection and analysis were performed by Naoaki Ito and Claudia Jean Kacmarcik. The first draft of the manuscript was written by Claudia Jean Kacmarcik, and all authors commented on previous versions of the manuscript. All authors read and approved the final manuscript.

## CONFLICT OF INTEREST STATEMENT

The authors declare no conflicts of interest.

## ETHICS STATEMENT

The University of Delaware Institutional Review Board approved this study (number: 1554102). Informed consent was obtained from all individual participants and/or legal guardians of participants under age 18 included in the study.

## Data Availability

The data that support the findings of this study are available from the corresponding author upon reasonable request.
